# HIV incidence trends in Brazil and neighboring countries: an ecological and analytical study on public health

**DOI:** 10.3389/fpubh.2025.1625475

**Published:** 2025-10-17

**Authors:** Thiago Augusto Ferreira dos Anjos, Aline Moraes Monteiro, Daniele Melo Sardinha, Luiza Raquel Tapajós Figueira, Marcos Jessé Abrahão Silva, Rebecca Lobato Marinho, Mayara Annanda Oliveira Neves Kimura, Tamires de Nazaré Soares, Luana Nepomuceno Gondim Costa Lima

**Affiliations:** ^1^Epidemiology and Public Health Surveillance Program of the Evandro Chagas Institute (PPGEVS/IEC/MS), School of Public Health of the University of São Paulo (FSP/USP), São Paulo, Brazil; ^2^Universidade do Estado do Pará/Hospital Ophyr Loyola (UEPA/HOL), Belém, Brazil; ^3^Postgraduate Program in Parasitic Biology in the Amazon, State University of Pará and Evandro Chagas Institute (PPGBPA/UEPA/IEC), Belém, Brazil; ^4^University of Amazonia (UNAMA), Ananindeua, Brazil; ^5^Pará State University (UEPA), Belém, Brazil; ^6^Bacteriology and Mycology Section, Molecular Biology Laboratory, Evandro Chagas Institute (SABMI/LABMOL/IEC), Ananindeua, Brazil; ^7^Postgraduate Program in Epidemiology and Health Surveillance, Evandro Chagas Institute (PPGEVS/IEC), Ananindeua, Brazil

**Keywords:** incidence, South America, indicators, public health surveillance, HIV infection

## Abstract

**Introduction:**

The Human Immunodeficiency Virus (HIV) is a global public health problem. In Latin America this problem is aggravating and widespread, with regard to the countries bordering Brazil, the notification of cases among men and women shows an aggravating panorama, which requires actions and services aimed at monitoring and changing this chain of transmission.

**Objective:**

To investigate the trend of HIV incidence in Brazil and neighboring countries from 2013 to 2023.

**Methods:**

An ecological, retrospective and quantitative study on HIV in Brazil and border countries, Excel 2019 and R language were used to process and analyze the data, it is worth noting that no data was found for French Guiana.

**Results and discussion:**

Based on the analyses performed, including violin statistics, linear regressions, ARIMA models, and comparability tests, it was possible to identify relevant patterns among the countries observed. Bolivia showed consistent and statistically significant growth in the HIV incidence rate, with an increase of 83.8% over the decade analyzed (R^2^ = 0.67; *p* = 0.0022). On the other hand, Guyana, although still among the countries with the highest incidence rates, showed a significant reduction of 31.6%. Suriname remained one of the countries with the highest rates throughout the period, exceeding 100 cases per 100,000 inhabitants in certain years. Spatial analysis revealed considerable disparities, particularly in border areas, where there is a higher concentration of cases, indicating the need for targeted public policies and shared surveillance efforts. Countries such as Peru, Colombia, and Paraguay also showed a growing trend, while Argentina, Uruguay, and Venezuela showed a decline or stagnation, although in some cases this apparent stability may be linked to underreporting.

**Conclusion:**

Some countries need to strengthen actions and services to monitor and break the chain of transmission, and implement public policies, as well as re-evaluate and ensure in countries with socio-economic and political crisis, in order to transform this aggravating panorama in Latin and South America.

## 1 Introduction

The HIV/aids (Human Immunodeficiency Virus/Human Immunodeficiency Syndrome) epidemic in Brazil had its first cases around the 1980s, before the enactment of the 1988 Federal Constitution and the regulation of the Unified Health System (SUS) through Federal Law 8.080/1990 (1). HIV infection and becoming ill with AIDS remain important public health problems in the world and in Brazil, having a significant impact on various segments of the population over the last few decades (2).

Brazil borders ten South American countries, several of which face high rates of HIV and share similar socio-epidemiological vulnerabilities. In regions near these borders, migratory movements, the integration of health systems, and common public health challenges make it essential to understand how the epidemic unfolds across neighboring territories (3).

According to UNAIDS (4), around 39.9 million people in the world were living with HIV in 2023. Of these, around 1.3 million represent new cases of HIV and 630,000 died from AIDS-related illnesses. Globally, in 2023, 9.2 million people living with HIV did not have access to antiretroviral therapy (ART), contributing to a significant number of preventable deaths over the year (5). In 2018 and 2019, 1.7 million new cases were registered worldwide, with a reduction to 1.5 million in 2020, of which 110,000 come from Latin American countries (6).

Pan American Health Organization (PAHO) data, the incidence of HIV in Latin America is estimated to have increased by 21% since 2010, with around 120,000 new cases in 2019. According to estimates by the PAHO, approximately 23% of people living with HIV in Latin America and the Caribbean are unaware of their serological status, highlighting significant challenges in expanding access to early diagnosis, with advanced AIDS. In 2019, around 1.3 million people in Latin America were taking ART, representing 60% of people living with HIV (7). The progress achieved over the years is the result of committed public policies and the search for strategies, especially international resolutions focusing on the most vulnerable populations (8). Therefore, the adoption of regional political strategies aligned with the vulnerable population of Latin America can be important factors in achieving goals.

Nationally, Brazil has recorded 1,165,599 AIDS cases since 1980, with an annual average of around 36,000 new cases in the years 2019 to 2024. Since 1980, 392,981 deaths from AIDS have been recorded in the country, of which 70.1% occurred among men and 29.9% among women, with a reduction in the mortality coefficient over the last 10 years of 32.9%, from 5.7 in 2013 to 3.9 deaths per 100,000 inhabitants in 2023 (2). In 2020 there was the largest annual reduction in the HIV detection rate, with around 14.1 cases per 100,000 inhabitants, which is related to underreporting caused by the overload of health services during the pandemic period (1).

In view of this, UNAIDS has established the 95–95-95 targets to end the global AIDS problem by 2030. These targets establish that 95% of people know their diagnosis, 95% of people with a confirmed diagnosis is on treatment and 95% of people living with HIV are on viral suppression (9). Despite this, Latin American countries need to devise efficient strategies to control the AIDS pandemic. Data from 2020 show that Brazil and Mexico were the countries that most complied with the recommendations of laws and policies aimed at HIV/AIDS with 79.6 and 74%, respectively, while those that least complied were Peru 30%, Venezuela 44.4% and Bolivia 44.4%, showing disparities between the achievement of UNAIDS targets (10).

Given this scenario, and considering that the collection and processing of epidemiological data can serve as a foundation for planning and implementing health actions, this study aims to analyze HIV trends in Brazil and neighboring countries between 2013 and 2023. The methods applied include violin plots, linear regression, ARIMA modeling, Joinpoint analysis, and spatial variation mapping. These approaches make it possible to identify shifts over time and across regions, offering a more precise and comprehensive interpretation of the epidemic than traditional summary measures.

## 2 Methods

### 2.1 Type of study

Ecological study, using public data, the data used in this study were extracted from the interactive portal of the Pan American Health Organization (PAHO), available at (Situação do HIV nas Américas - OPAS/OMS | Organização Pan-Americana da Saúde), which compiles information submitted by member countries and estimates from UNAIDS. The historical records access data from 2013 to 2023; the year 2024 was not collected because there was no recorded data, so the time frame was limited to 10 years. In the case of Brazil, data from the Notifiable Diseases Information System (SINAN) were also used only in 2023, as there were no data for that year in 2023 on Brazil. SINAN is one of Brazil’s main national epidemiological surveillance databases, which reports on various diseases, including HIV/AIDS cases. The statistics reflect official notifications of new cases by country, and incidence calculations were based on the estimated annual population, according to Worldometer data. All of these sources were used to ensure the traceability and authenticity of the information.

### 2.2 Location and population

**Figure 1:**
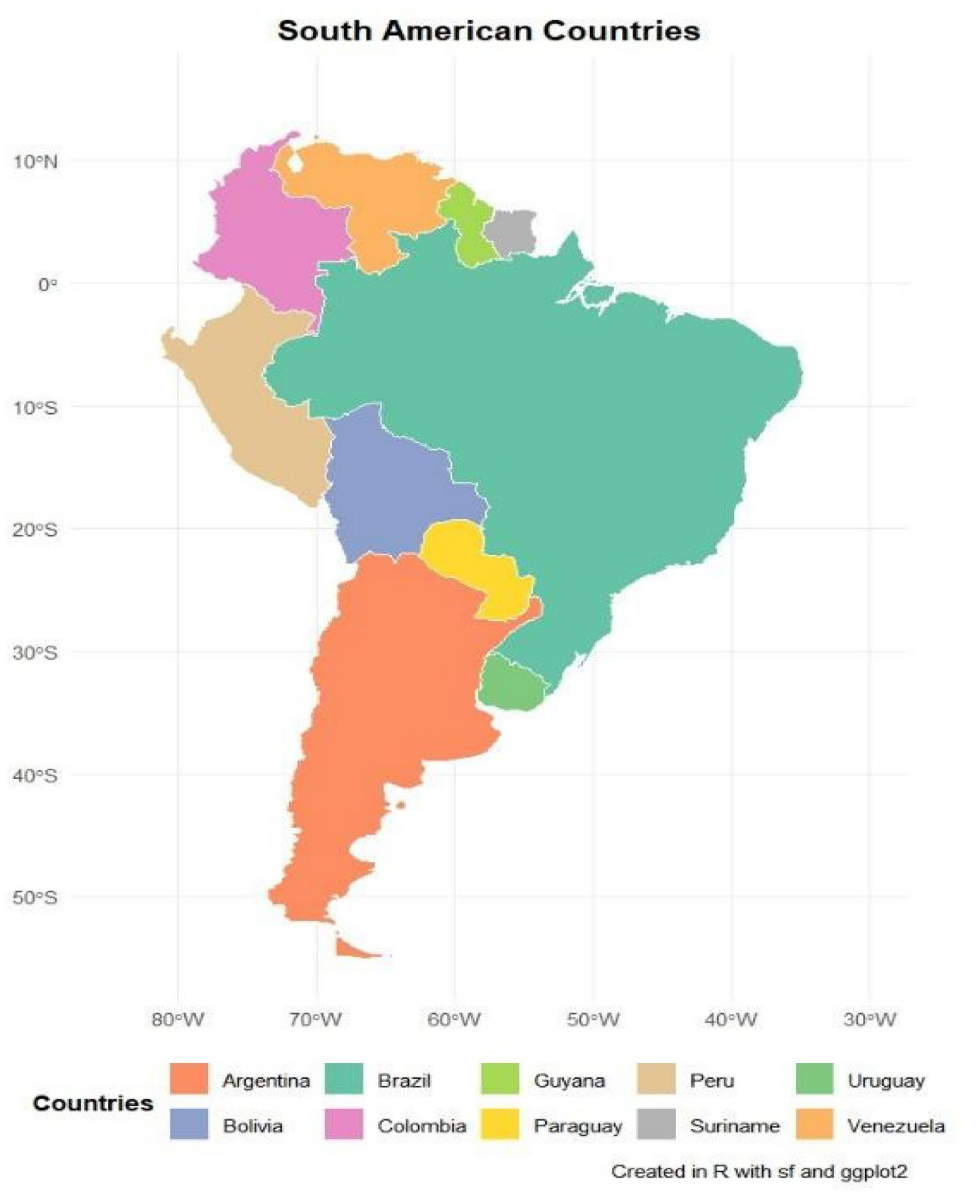
Geographical distribution of the countries that border Brazil and are part of the HIV survey among men and women. Source: dos Anjos et al. (30).

The study stems from another study focused on HIV and aids research in Brazil. In this research in Latin America, HIV cases among men and women were evaluated in Brazil, a country located in eastern South America, on the border with the Atlantic Ocean, with a total area of 8,515,770 km, with a total population of around 220,051,512 people; Bolivia, located in Central South America in the southwest of Brazil, has a total area of 1,098,584 km and a population of around 12,311,974 people. 64 098,581 km and a population of around 12,311,974; Colombia located in the North of South America, with a total area of 1,138,910 km and a population of 49,588,357, Peru in the West of South America, with an area of 1,285,216 km and a total population of 32,600. 249 people; Paraguay, located in Central South America, has an area of 406,752 km and a population of around 7,522,549; Argentina, located in Southern South America, has an area of 2,780,400 km and an estimated population of 46,994,384; Guyana, in Northern South America, has an estimated population of 794,099 and a total area of 214. 70 969 km; Suriname, located in the North of South America, has an area of 163,820 km and an estimated population of 646,758 people, Venezuela in the North of South America, with an area of 912,050 km and a population of 31,250,306 people and Uruguay in the South of South America, with an area of 176,215 km and a population of 3,425,330 people, according to Figure 1. No HIV data was found in French Guiana (11).

### 2.3 Research variables and tabulation

The primary variables in this study were new HIV case notifications among men and women, grouped by country of residence. Data were collected and organized using Excel 2019. Due to limitations in public datasets from official platforms, analysis was conducted in aggregate form, without age stratification. The terms “men” and “women” refer to biological sex as recorded in national epidemiological surveillance systems.

When comparing HIV incidence across countries, age-standardized rates were not applied because of restricted access to disaggregated population data. This limitation, which may affect comparability between nations with markedly different demographic profiles, has been acknowledged in the interpretation of findings.

### 2.4 Statistical analysis

The analysis was carried out in the R language, first presenting Table 1 with data collected directly from the World Health Organization (WHO) website, another table with the incidence rate, the calculation of the incidence rate was carried out per 100,000 inhabitants according to PAHO parameters, for the calculation per year, the total population per year was taken from the Covid monitoring website in the world (COVID - Estatísticas do Coronavírus - Worldometer). Violin statistics help to characterize the descriptive information, because the more expanded the violin is, the greater the concentration of cases, showing where the number of cases is between certain years, as well as a boxplot with the mean, median and quartiles of cases, which helps to better characterize the research, as for the linear regression in a single visualization, it helps to show where the trend in HIV between 2013 and 2023 was increasing, stagnant or decreasing, the regression has the trend coefficient which shows how much it grew per year, the coefficient of determination which shows how well the values fit the regression and the *p*-value, indicating whether this trend is significant. The Arima model with time series was created to show where there was growth, reduction and stagnation over periods of years, providing a better epidemiological mapping of the aggravating factor, while the sophisticated Joinpoint analysis shows in which year the change in trend occurred, in addition to presenting the state of this trend, the Tukey test of comparability presents the comparison in the detection rate between countries, showing the difference by country, Heatmap or heat map showing the concentration by year and Heatmap 2 with dendrogram showing the similarity of cases by year and by country, thus mapping the panorama of HIV among men and women in these countries and finally the spatial analysis bringing the percentage variation of the first year 2013 and 2023 and showing whether there has been growth, reduction or stagnation of cases in the last 10 years and how the percentage is in relation to this temporality.

**Table 1:** Numbers of HIV cases in men and women in Brazil and countries bordering the South American country.

Year	Brazil	Argentina	Bolivia	Colombia	Guyana	Paraguay	Peru	Suriname	Uruguay	Venezuela
2013	43.269	6.900	1.779	9.124	833	1.207	5.570	470	1.060	-
2014	42.122	6.053	2.066	9.719	597	1.369	6.352	-	1.016	6.131
2015	40.649	5.974	2.291	8.209	705	1.430	7.597	511	941	5.684
2016	39.107	5.228	2.445	9.399	855	1.441	7.418	-	856	7.882
2017	37.791	4.567	2.962	10.246	961	1.443	6.554	-	797	6.547
2018	38.251	4.581	3.184	10.930	772	1.564	8.074	597	987	4.012
2019	37.731	3.937	3.161	12.528	807	1.605	9.422	598	993	4.052
2020	37.023	2.996	2.490	9.210	293	1.201	5.872	414	876	4.970
2021	42.676	5.685	2.985	12.919	590	1.318	8.542	552	757	-
2022	43.043	6.073	3.417	14.670	459	1.519	9.096	598	867	-
2023	46.495	6.588	3.748	-	628	1.651	10.008	636	870	-

Source: World Health Organization (7).

### 2.5 Packages used in the analysis

Statistical analyses were performed using R version 4.4.2. The following packages were used for each type of analysis:

*For violin plots*: ggplot2, readxl, and tidyverse were used.
*Linear regression*: Readxl, dplyr, tidyr, broom, ggplot2, gridExtra, and Purr.
*ARIMA models*: forecast, TSA, and tseries.
*Joinpoint regression*: segmented, dplyr, patchwork.
*Tukey test (post-hoc)*: dplyr, tidyr, broom, and gridExtra.
*Heatmaps*: pheatmap, RColorBrewer, dplyr, tidyr, readr, data, table, scales, and base.
*Spatial analysis with variation*: Pacman, sf, tidyverse, RColorBrewer, and patchwork.

### 2.6 Formulas used to calculate incidence and variation

To calculate the incidence rate, the following formula was used to multiply it by 100,000 inhabitants:


$$\text{Incidence}=(\frac{\text{Number of}\;\mathrm{new}\;\text{cases during the period}}{\text{Population}\;\mathrm{at}\;\text{risk during the same period}})\times 10^{7}$$

The following formula was used for the percentage change:


$$\text{Percentage Change}=(\frac{\text{Final Value}-\text{Initial Value}}{\text{Initial Value}})\times 100$$

### 2.7 Research ethics committee

The study did not directly or indirectly involve studies with human beings or the identification of names and addresses. Thus, submission to the ethics committee is unnecessary, since the data is in the public domain, available on the WHO website and in the Notifiable Diseases Information System (SINAN- Brazil) in 2023.

## 3 Results

In the temporal analysis of the last decade in Brazil and in the countries that border it, the lack of notification of cases was a recurring factor, especially in Venezuela, Suriname, noting the lack of notification on the site in 2023 in Colombia, in a second analysis, the countries with the highest numbers of notifications in the series evaluated were Brazil, Colombia, Peru, Argentina, Bolivia and Uruguay (Table 1).

In the incidence rate, all calculations were made per 100,000 inhabitants and divided by the total population per year, Guyana in 2013 had 111.07 new cases per 100,000 inhabitants, in 2023 there were 76.01 cases per 100,000 inhabitants (inhab), Suriname in 2013 had 82.69/100,000 inhab, in 2023 it was 101. 15/100,000, being the two countries with the highest incidence rates in the survey, then Colombia showed a growth in 2013 it was 19.76/100,000 inhab, in 2022 it went to 28.36/100,000 inhab, Bolivia and Peru showed the same characterization of growth, as shown in Table 2.

**Table 2:** HIV incidence rate among men and women in countries bordering Brazil, with high cases in Guyana, Suriname and increases in countries such as Bolivia, Colombia and Peru.

Year	Brazil	Argentina	Bolivia	Colombia	Guyana	Paraguay	Peru	Suriname	Uruguay	Venezuela
2013	21.52	16.20	16.65	19.76	111.07	20.18	18.69	82.59	31.69	-
2014	20.77	14.07	19.04	20.87	79.16	22.56	21.10	-	30.27	20.26
2015	19.88	13.74	20.80	17.47	92.92	23.22	24.95	87.85	27.95	18.59
2016	18.97	11.91	21.87	19.82	111.96	23.05	24.03	-	25.33	25.62
2017	18.20	10.31	26.10	21.29	125.03	22.76	20.92	-	23.52	21.42
2018	18.35	10.26	27.66	22.29	97.55	24.33	25.31	99.61	29.07	13.46
2019	17.96	8.75	27.09	25.11	99.91	24.63	29.04	98.71	29.23	14
2020	17.48	6.63	21.07	18.19	36.28	18.19	17.88	67.61	25.78	17.47
2021	19.99	12.54	25.00	25.23	72.35	19.72	25.76	89.34	22.29	-
2022	19.99	13.38	28.29	28.36	55.86	22.46	27.17	95.97	25.57	-
2023	21.88	14.47	30.61	-	76.01	24.12	29.56	101.15	25.68	-

Source: dos Anjos et al. (30). Data from the World Health Organization (WHO).

**GRAPH 1:**
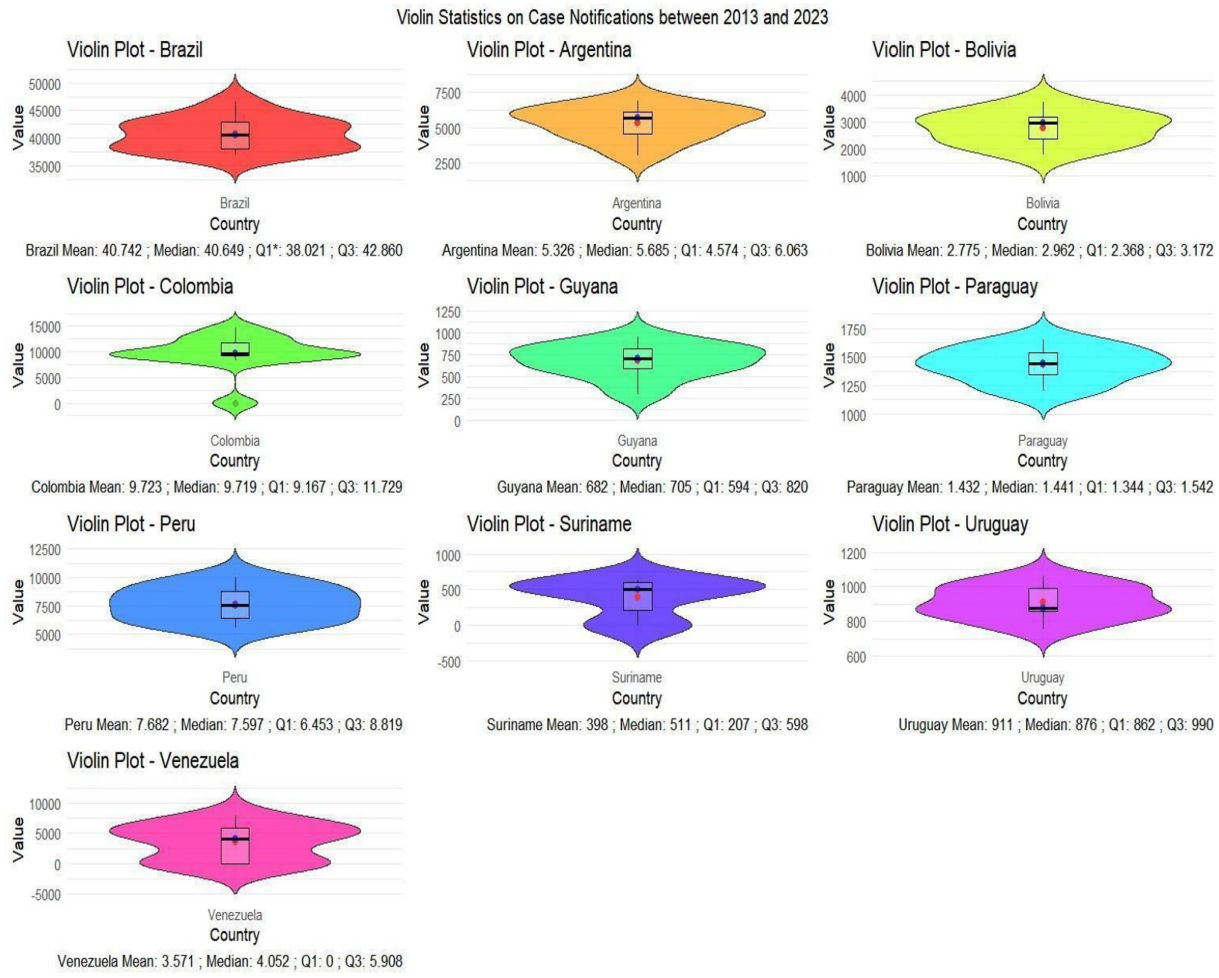
Violin statistics with concentration in certain case intervals, as well as the creation of a box plot showing the mean, median and standard deviation within the 2013–2023 timeframe. Source: dos Anjos et al. (30). Data from the World Health Organization (WHO).

Regarding new HIV cases, the violin statistics showed details and characteristics in each country, the characterization of the violin shows the variability of cases in different countries, Brazil had a concentration of cases between 40,000 and 45,000 cases between 2013 and 2023, with the highest average at 40,742 and median at 40. 649, while the concentration of cases in Colombia was between 10,000 and 15,000 cases, with the second highest average at 9,723 and median at 9,719 cases, followed by Peru with a concentration between 7,500 and 10,000 cases, average of 7,682 and median at 7,597, followed by Bolivia with a concentration of cases between 2,000 and 4,000, with an average of 2,775 and a median of 2,962 (Graph 1).

**GRAPH 2:**
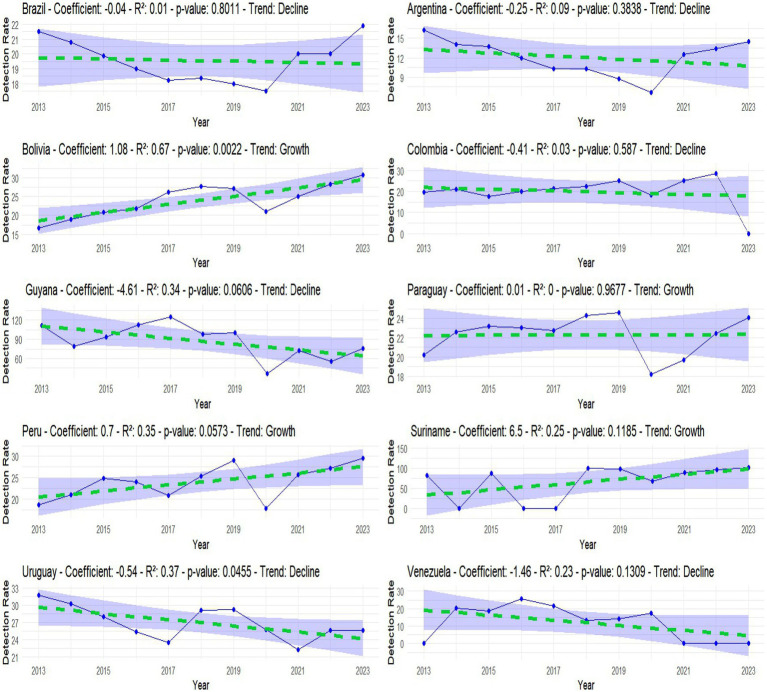
Linear regression showing whether there was a reduction, stagnation or growth in cases between 2013 and 2023. Source: dos Anjos et al. (30). Data from the World Health Organization (WHO).

In the linear regression analysis, data from 2013 to 2023 was plotted, with treatments for the years with no data. In this 10-year time analysis, Bolivia showed an increase in the average rate per year of 1.08 units, and the coefficient of determination and *p*-value confirm this growth in HIV, and the regression line was adjusted in order to adjust the margin of error and precision of the data. In Brazil, Guyana, Uruguay and Venezuela there was a reduction in cases, Colombia and Peru showed stagnation in cases with R2 very close to 0, Peru and Suriname showed an increase in cases (Graph 2).

**GRAPH 3:**
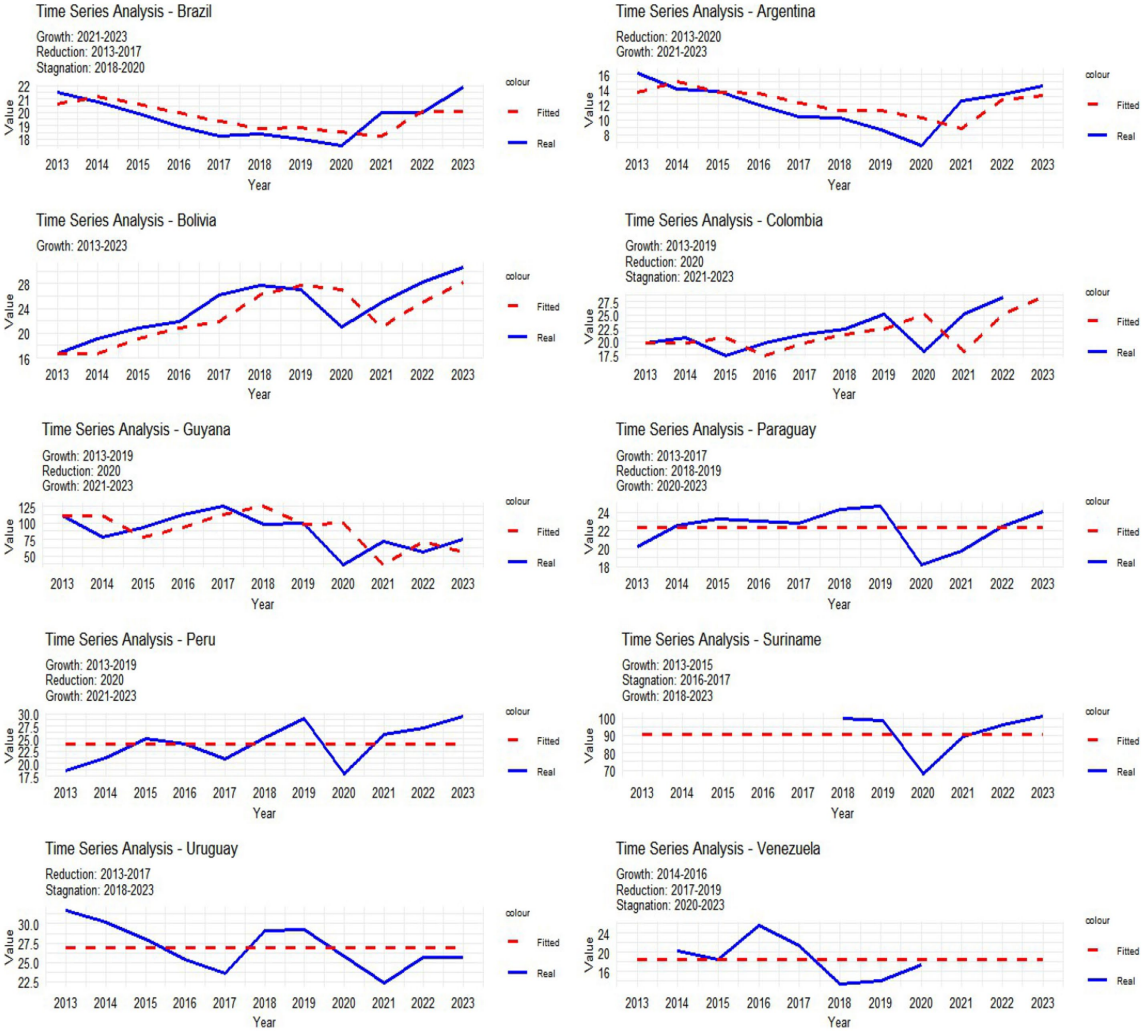
Simple Arima complementing image 2, on growth, stagnation or reduction of HIV cases between 2013 and 2023. Source: dos Anjos et al. (30). Data from the World Health Organization (WHO).

The linear regression model was important in analyzing the data, providing analytical information on the incidence rate, even with adjustments of 95%, but the vast majority of R2s were small, aiming at an in-depth analysis of this 10-year period, a simple Arima model was performed, analyzing the data, It can be seen that this was done using real and adjusted data and, based on this, it was shown whether the evolution of cases was increasing, stagnating or decreasing over a given period of time, for example from 2013 to 2017 there was a reduction in the incidence rate in Brazil, 2018–2020 stagnation, and 2021–2023 there was growth, this analysis covers the other countries, as shown in the image below (Graph 3).

**GRAPH 4:**
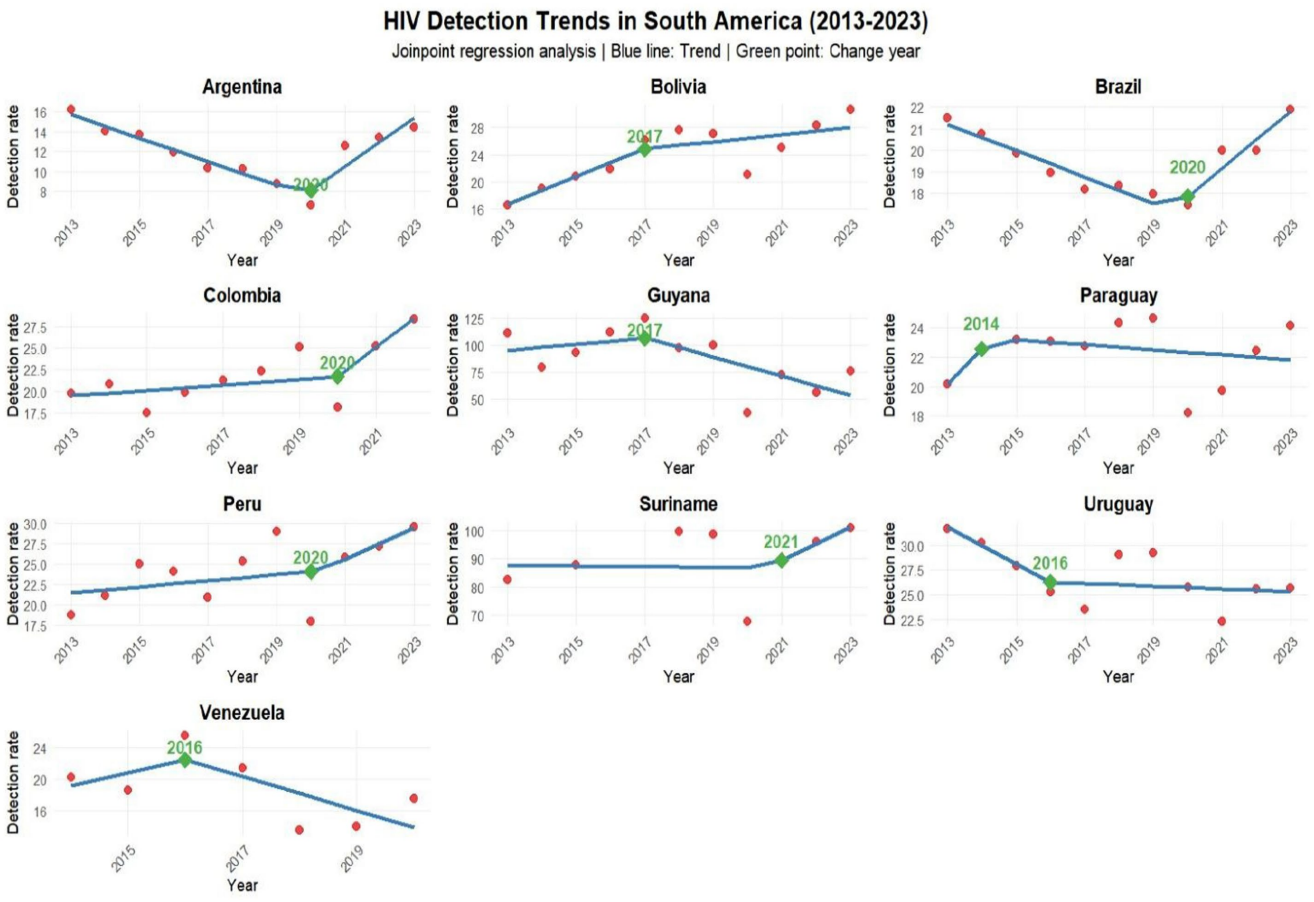
Joinpoint analysis showing in which year the change in trend occurred in each country bordering Brazil. Source: dos Anjos et al. (30). Data from the World Health Organization (WHO).

The Joinpoint analysis describes where the trend changed, an important analysis in the research, as it shows in which year the change took place. In Argentina, Brazil, Colombia and Peru this change in trend occurred in 2020, in Bolivia and Guyana in 2017, Paraguay in 2014, Suriname 2021, Uruguay and Venezuela in 2016, as shown in Graph 4.

**Figure 2:**
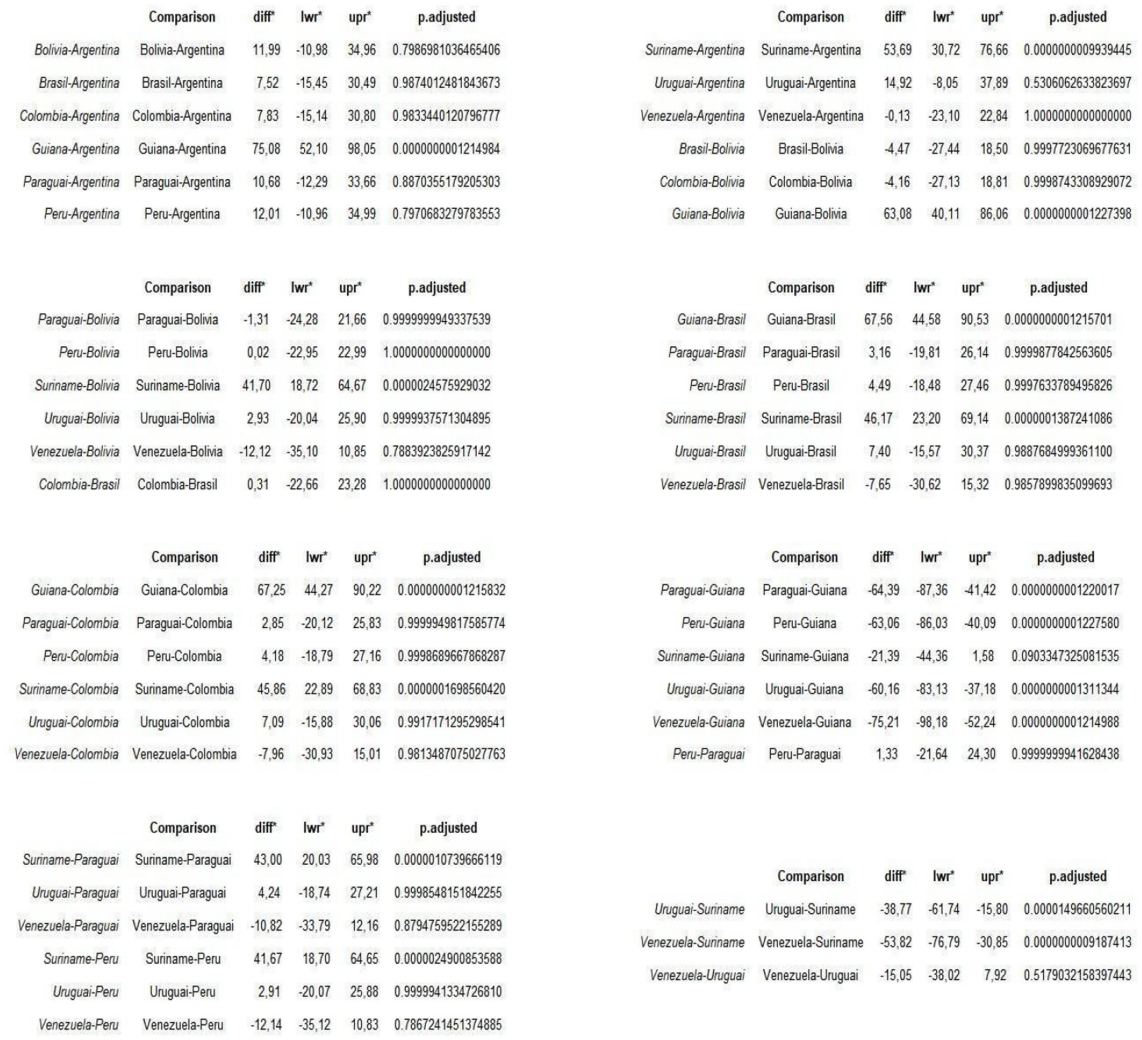
Comparative analysis of incidence rates between countries bordering Brazil. Source: dos Anjos et al. (30). Data from the World Health Organization (WHO). *Diff, Difference. *Lwr, Lower limit. *Upr, Upper limit.

The Tukey test showed the difference in the incidence rate between countries, the diff represents the difference, the lower limit of the confidence interval for the difference (lwr), the upper limit, indicating that this value is between the upper and lower limit (upr), in addition the adjusted *p*-value indicates whether this difference is significant, the comparisons were made and the statistical analysis, in all comparisons the vast majority gave a *p*-value above 0.05, not refuting the null hypothesis and confirming that the diff there really is a difference. On the other hand, most of the countries whose incidence rates were compared with Suriname and Guyana had a *p*-value below 0.05, indicating that this difference is significant, especially in the two countries mentioned compared to their neighbors. Positive diff values indicate that the first country has a higher rate compared to the second, while negative values indicate that the second has a higher rate, for example, comparison between Guyana and Argentina (*p*-value 0.00000000012), with a significant *p*-value, showing that the difference is 75.08 cases higher in Guyana, while between Suriname and Bolivia (*p*-value 0.0000024), this difference was significant for Suriname with a difference of 41.70 cases; in Guyana and Colombia (*p*- value 0.00000000012) this difference was 67.25 for Guyana, all the countries that were compared with Guyana and Suriname, the difference was higher in these countries, as shown in Figure 2.

**MAP 1:**
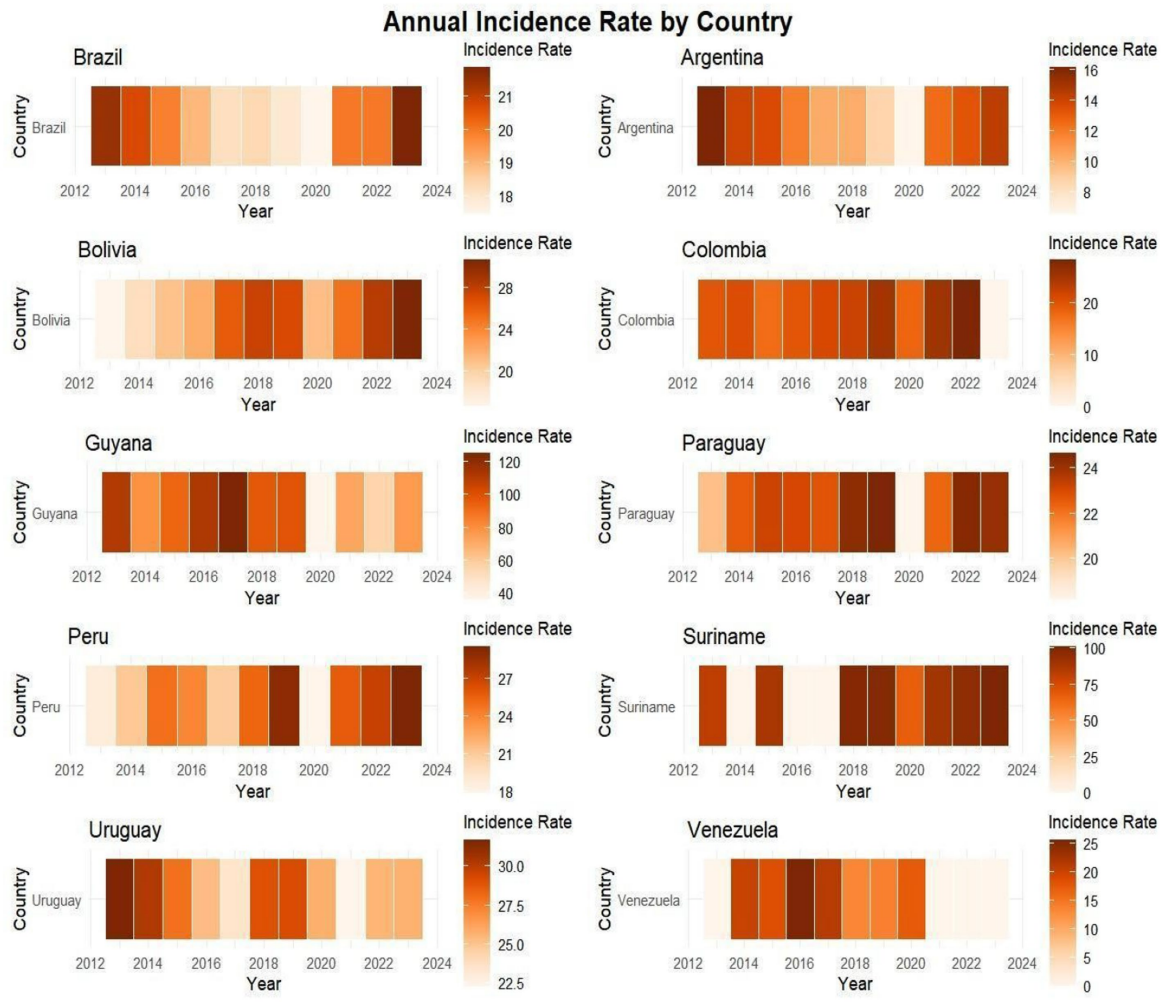
Heatmap showing the intensity of cases by heatmap in the countries described between 2013 and 2023. Source: dos Anjos et al. (30). Data from the World Health Organization (WHO).

In the heatmap, the analysis showed in which year the highest rates of new cases were concentrated, the highest incidence rates are concentrated in Guyana with a concentration between 40 and 120 cases and in Suriname ranging from 0 to 100 cases, with values exceeding 100 cases per 100,000 inhabitants, some blank gaps, as there was no notification in these countries, such as Venezuela in 2021 to 2023 and Suriname 2014, 2016 and 2017. In addition, Bolivia between 20 and 24 cases, Uruguay between 22.5 and 30.00, Colombia with 0 to 30 and Brazil between 18 and 21 showed significant concentration rates. The higher the concentration of color, the higher the incidence rate per year, as shown in the image below (Map 1).

**MAP 2:**
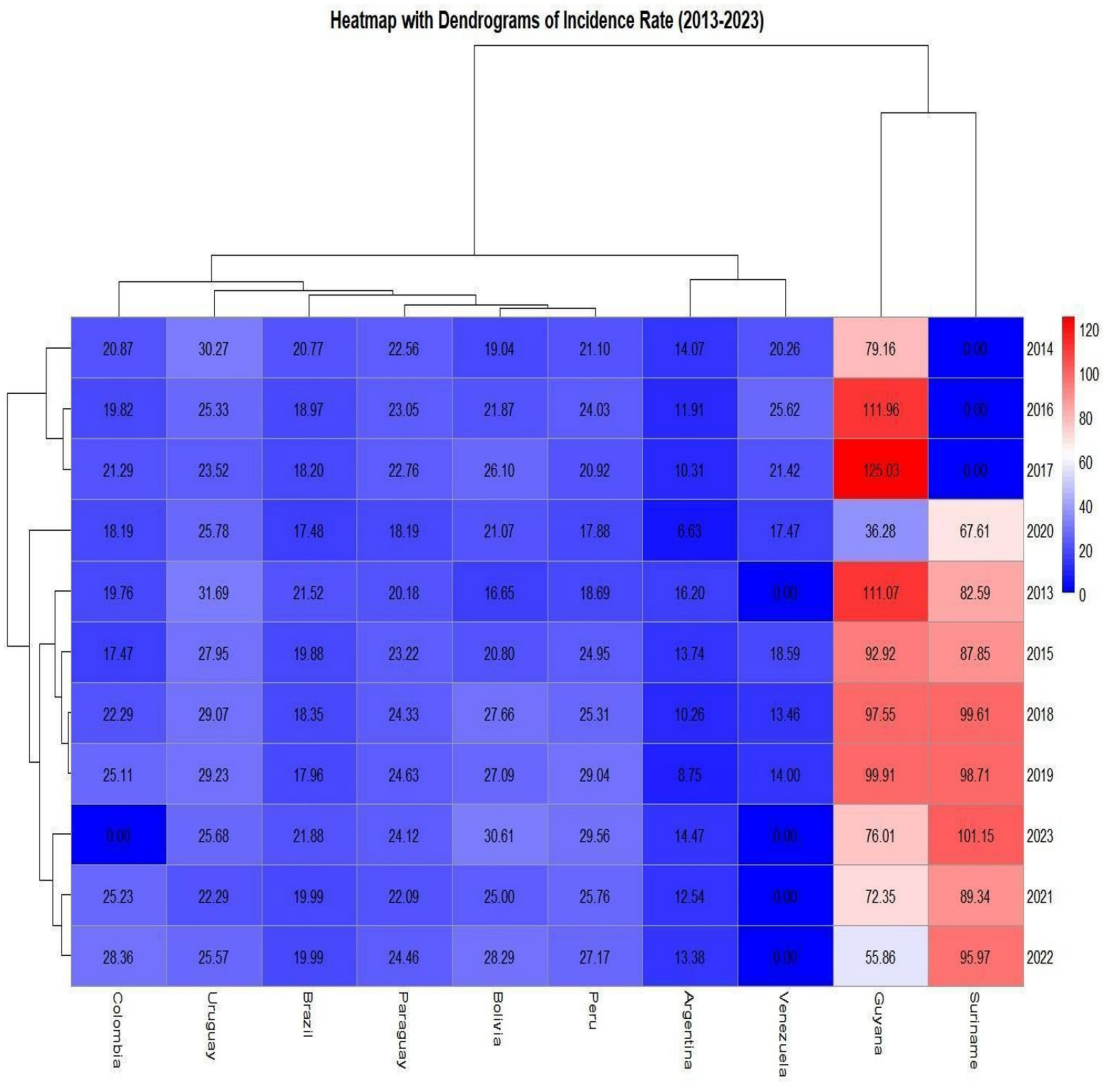
Heatmap with dendrogram showing which countries had similar incidence rates between 2013 and 2023. Source: dos Anjos et al. (30). Data from the World Health Organization (WHO).

In the heatmap with description and grouping of countries based on similarity of incidence rate, once again Guyana and Suriname had a high intensity according to Map 2 with 50 to 100 cases, here the dendrogram or cluster tree, showed that the two countries in most years had similarity in the HIV rate. On the vertical axis, Uruguay, Paraguay, Bolivia, Brazil, Colombia and Peru had similar incidence rates both vertically and horizontally, representing the rates per year; Argentina and Venezuela had some similar rates in certain years, the closer the cluster tree was to each other, In Suriname and Guyana, although the tree shows that certain years are similar, the tree is a little distant, because some years there were no notifications in Suriname and other rates are not similar (Map 2).

**MAP 3:**
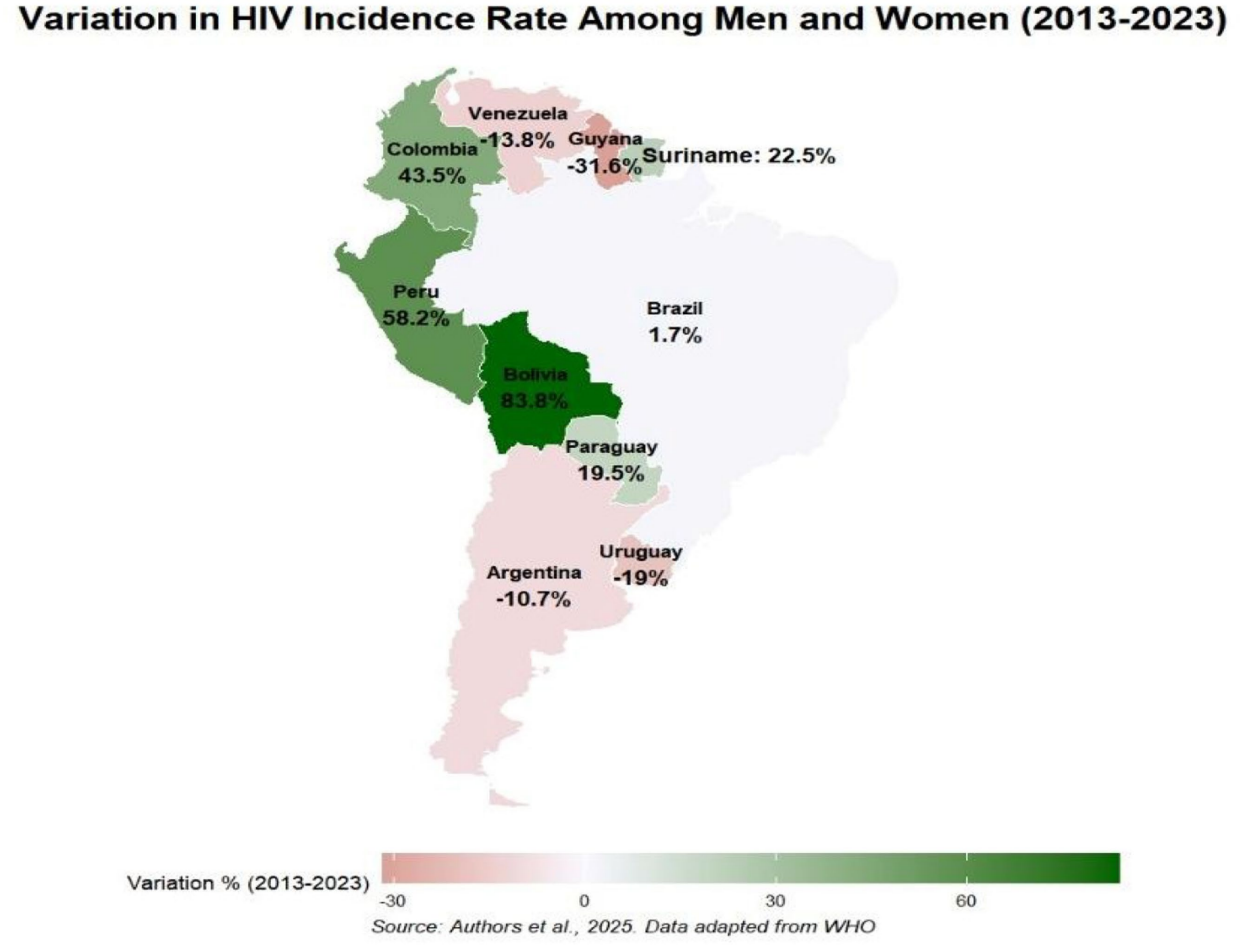
Variation in HIV among men and women in Latin American countries. Source: dos Anjos et al. (30). Data from the World Health Organization (WHO).

In terms of the variation in the incidence rate, the country that has seen an increase in the incidence rate over the last 10 years is Bolivia, with an increase of 83.8% compared to the reference point of 2013, Peru also showed a worrying increase of 58.2%, Colombia a significant increase of 43.5%, Paraguay with a small increase of 19.5% and Suriname at 22.5%. Brazil had a slight drop of 1.7%, countries such as Argentina with −10.7%; Uruguay −19% showed a small drop in the number of cases, unlike Guyana with a significant drop of −31.6% in the number of cases, although Guyana has a high HIV rate, the drop in recent years has been significant and finally Venezuela with −13.8%, this reduction in cases in Venezuela may be related to factors such as underreporting of cases, since it is the country with the highest number of underreported cases. The variation in cases in Colombia was made with data from 2013 (19.76) and 2022 (28.36), Venezuela with 2014 (20.26) and 2020 (17.47), as shown in Map 3.

## 4 Discussion

The study showed that the epidemiological panorama for each country had a significant difference, where countries like Uruguay, Argentina and Guyana showed a reduction in HIV cases over the 10- year period, while the others showed a slight increase, Brazil. This difference may be linked to factors such as political, economic and technological action in tackling the virus in the respective countries, the absence of data in some years suggests internal precariousness in the follow-up and monitoring of data, in Venezuela in addition to the political crisis that interferes in all sectors of society, the lack of resources makes it difficult to obtain information and monitor virology, in Suriname with precarious integration with the rest of its neighbors and social inequalities, data analysis can become a significant problem (12, 13).

In the violin statistics, the data reveal the approach adopted by each country over the past 10 years, with Brazil showing the highest average and median number of cases among men and women, followed by Colombia and Argentina. Brazil is not only the largest country, but also holds the largest population in South America compared to its neighbors. In this context, it is important to highlight a recent initiative that may positively influence national indicators: the *Programa Brasil Saudável* (Healthy Brazil Program), launched in 2024 by the Federal Government. This interministerial strategy is aimed at eliminating socially determined diseases, including HIV/AIDS. The program promotes integrated actions across health, education, social assistance, and infrastructure sectors, with special focus on vulnerable populations and priority territories. Although results are still in the early stages of measurement, the initiative has already shown meaningful progress, including expanded diagnostic coverage and enhanced epidemiological surveillance. The inclusion of HIV among the target diseases reinforces Brazil’s commitment to increasing access to diagnosis, treatment, and the fight against vertical transmission, in line with the UN 2030 Agenda and the guidelines of the Pan American Health Organization. Regarding Guyana, although this country has one of the highest detection rates, its average number of cases in the violin statistics was lower than Venezuela’s, which indicates greater consistency and effectiveness of health services in Guyana. This panoramic view of the countries analyzed underscores how the implementation and strengthening of HIV/AIDS public policies are reflected in society. Greater program implementation tends to lead to broader availability of testing, diagnosis, and case detection. However, the fragility of health education continues to contribute directly to rising case numbers in these regions (14–17).

In the linear regression analysis, Bolivia was the only country with a significant trend coefficient, coefficient of determination, and *p*-value, showing an upward trend over the last 10 years. According to the World Health Organization, Bolivia is one of only three Latin American countries that increased spending on healthcare services. Thus, this high rate may be linked to reforms in Bolivia’s unified public health system model, including greater investments in infrastructure, equipment, and human resources to serve both the general population and vulnerable groups. The improvements in HIV- related services have been so significant that the country is now proposing amendments to its HIV law, aiming to provide comprehensive and free treatment for all people living with HIV/AIDS (18, 19).

Most countries showed a significant decline in 2020 according to linear regression, ARIMA, and joinpoint models, driven by the COVID-19 pandemic, which diverted most public health services toward COVID-19 response. However, countries like Brazil, Argentina, Peru, Suriname, Guyana, Paraguay, and Bolivia experienced an increase in new case rates between 2021 and 2023. This trend may be linked to improved reporting, enhanced healthcare services, public awareness campaigns, and health education initiatives. Nevertheless, country-specific economic, political, and social factors likely influenced these rates. For instance, Argentina’s economic crisis may significantly impact its HIV/AIDS public policies—a major public health concern. Similarly, data reporting deficiencies in Venezuela and Suriname raise alarms: the former faces socioeconomic and political conflicts that directly disrupt healthcare services and data reporting, while the latter suffers not only from healthcare system limitations but also from stigma and insufficient investment in public policies (20–23).

In addition to internal factors such as economic instability, cross-border dynamics also play a significant role in the epidemiological landscape. In border regions, particularly those involving Brazil, Colombia, and Venezuela, initiatives such as the UNAIDS Joint Program, technical missions from the Pan American Health Organization (PAHO), and regional pilot projects have been implemented to expand access to PrEP, with a focus on HIV prevention and testing. These actions include the provision of self-testing kits, mobile initiatives targeting key populations, and community education in transit centers such as Pacaraima (Brazil) and Cúcuta (Colombia), which receive a large influx of displaced persons. However, significant challenges remain, such as migratory pressures, treatment discontinuity, and sociocultural factors such as stigma, risky behaviors, language barriers, and more conservative political agendas, observed in countries such as Paraguay, Venezuela, and Argentina, which may limit the implementation of preventive strategies, especially those focused on sex education, care, treatment, HIV/AIDS programs, and stigma reduction. This scenario can compromise data consistency and undermine the effectiveness of long-term interventions in public health and society (7–26).

In the Tukey comparability test, the comparison was significant primarily with Suriname and Guyana, which showed the highest incidence rates. As comparison profiles, the countries compared with Guyana and Suriname demonstrate the aggravated public health situation in these two nations. The other countries have populations counted in millions, while Guyana and Suriname, in thousands - and even in this accounting, they have the highest HIV rates in Latin America. In Heatmap 1, valuable insights about Guyana and Suriname were described: the high case numbers may be related to factors such as population mobility, poor infrastructure and public information systems (including for young adult groups), migration, and difficulties in implementing HIV/AIDS public policies. In this regard, the implementation and effectiveness of public policies, interventions, healthcare investments, education, and healthcare access are factors that need to be reanalyzed and strengthened in these countries (23, 27, 28).

Important insights in Map 2 showed the distribution of HIV cases, where the dendrogram revealed countries with certain similarities in cases between men and women. Again, Suriname and Guyana showed similarity in incidence rates throughout most of the 10-year period, highlighting the public health severity in these countries. Most countries - including Colombia, Uruguay, Brazil, Paraguay, Peru, and Bolivia - displayed similarities between 2013 and 2023. Colombia, Uruguay, Peru, and Bolivia showed case surges in certain years on the heatmap. Argentina and Venezuela exhibited similar characteristics. The blue colors indicate that the rates are not as significant or high. Related factors may include: effective program implementation, healthcare service investments, continuous education, testing availability, outreach to vulnerable groups, and expanded healthcare coverage - all potential contributors to these similarity patterns across Latin America. It’s crucial to note that underreporting in some countries like Venezuela and Suriname may not reflect the true reality, and Argentina’s political crisis could reshape the HIV/aids landscape in Argentine society (8, 9, 12–21).

In the variation of incidence rates, Guyana was the country with the greatest drop in case numbers. Our study achieved a positive result about Guyana’s epidemiological situation with a reduction of −31.6%, which reflects targeted actions such as test availability, improvements in care, treatment and health investments that may have led to the reduction in recent years. However, it’s important to emphasize that improvements in the HIV situation do not reduce the need for monitoring the problem, since Guyana has one of the highest adult prevalence rates in Latin America and the Caribbean according to data from the United Nations HIV/AIDS Program (UNAIDS). Bolivia, Peru, Colombia and Suriname should have greater availability of targeted actions for society and vulnerable groups, which includes improvements in health structures, strengthening and investments in programs, professional training and intensification of continuous and permanent education (8–29).

The absence of data from some countries was a limiting factor. Incomplete records and underreporting also represented research limitations, as some years (like 2023 and 2024) were not updated. Thus, the study’s limitations - including missing data and reporting gaps - mean that while using publicly available databases, the results may contain registration biases, outdated information, and lack of transparency, potentially presenting a different reality from each country’s epidemiological surveillance. However, the data were accurately processed to ensure research quality in this study. Finally, we reiterate the need for further studies, given that this article addresses general HIV data among men and women without stratification by age, as the WHO database is limited to general data only. We therefore highlight the need for complementary analyses with adjusted rates in future studies.

Regarding benefits, this study provides a distribution analysis of new HIV cases among men and women across Latin America and Brazil’s neighboring countries. It represents one of the first - if not the first - articles to conduct an in-depth analysis extending beyond Brazil to encompass other Latin American nations, offering valuable insights for public health and the quality of surveillance and public policy implementation in each country’s specific context. All data were collected directly from the World Health Organization (WHO) website.

## 5 Conclusion

Understanding the HIV epidemiological situation is crucial for promoting prevention and quality healthcare for the population. This study, conducted in Latin American countries bordering Brazil, revealed a notable pattern in HIV trends among men and women, showing significant growth after the Covid-19 pandemic according to joinpoint analysis, which provides higher accuracy assessment. Although Guyana has high incidence, the trend has been decreasing since 2017 as shown by joinpoint and spatial variation analysis, indicating that despite its high HIV rate, the pattern is one of reduction. The study also provides information about Suriname and Guyana, highlighting the importance of directing actions and services to evaluate interventions and implement effective public policies in these countries facing significant HIV challenges.

Another notable finding concerns Bolivia, which showed an increase in incidence rates compared to other countries. Factors linked to this include improvements and investments in Bolivia’s healthcare services, reaching more people and vulnerable groups, as well as greater implementation and strengthening of HIV/AIDS public policies. Brazil, the most populous country in South America, has shown a pattern of slight case growth. As a large nation with heterogeneous case distribution across its regions and federal units, its HIV/AIDS public policies should be analyzed and strengthened to improve monitoring of the epidemic.

An important finding relates to Venezuela. Despite missing data - which poses challenges for assessing public health quality and transparency in the country - Venezuela displayed case patterns similar to Argentina from 2013 to 2023. Other countries showed certain similarities according to heatmap 2, revealing comparable patterns in HIV/AIDS policy implementation and effectiveness, with each country demonstrating its own management approach and population coverage.

Regarding HIV/aids public policies, these remain critically important. Many countries currently face challenges including lack of reporting, rising case numbers, and persistent high incidence rates over multiple years. This situation highlights the essential role of public policies in monitoring, prevention, research, education and achieving targets - especially since the World Health Organization and Pan American Health Organization (WHO/PAHO) aim to eliminate AIDS as a public health problem in Latin America by 2030, a goal directly dependent on effective HIV monitoring.

## Data Availability

The original data presented in the study are publicly available. This data can be found here: https://www.paho.org/en/topics/hivaids and https://unaids.org.br/estatisticas/.
